# Modelling the suppression of a malaria vector using a CRISPR-Cas9 gene drive to reduce female fertility

**DOI:** 10.1186/s12915-020-00834-z

**Published:** 2020-08-11

**Authors:** Ace R. North, Austin Burt, H. Charles J. Godfray

**Affiliations:** 1 Department of Zoology, University of Oxford, Oxford, UK; 2 Imperial College, London, UK; 3 Oxford Martin School, University of Oxford, Oxford, UK

**Keywords:** Malaria, CRISPR-Cas9, Gene drive, Mosquito

## Abstract

**Background:**

Gene drives based on CRISPR-Cas9 technology are increasingly being considered as tools for reducing the capacity of mosquito populations to transmit malaria, and one of the most promising options is driving endonuclease genes that reduce the fertility of female mosquitoes. In particular, there is much interest in constructs that target the conserved mosquito doublesex (*dsx*) gene such that the emergence of functional drive-resistant alleles is unlikely. Proof of principle that these constructs can lead to substantial population suppression has been obtained in population cages, and they are being evaluated for use in sub-Saharan Africa. Here, we use simulation modelling to understand the factors affecting the spread of this type of gene drive over a one million-square kilometre area of West Africa containing substantial environmental and social heterogeneity.

**Results:**

We found that a driving endonuclease gene targeting female fertility could lead to substantial reductions in malaria vector populations on a regional scale. The exact level of suppression is influenced by additional fitness costs of the transgene such as the somatic expression of Cas9, and its deposition in sperm or eggs leading to damage to the zygote. In the absence of these costs, or of emergent drive-resistant alleles that restore female fertility, population suppression across the study area is predicted to stabilise at ~ 95% 4 years after releases commence. Small additional fitness costs do not greatly affect levels of suppression, though if the fertility of females whose offspring transmit the construct drops by more than ~ 40%, then population suppression is much less efficient. We show the suppression potential of a drive allele with high fitness costs can be enhanced by engineering it also to express male bias in the progeny of transgenic males. Irrespective of the strength of the drive allele, the spatial model predicts somewhat less suppression than equivalent non-spatial models, in particular in highly seasonal regions where dry season stochasticity reduces drive efficiency. We explored the robustness of these results to uncertainties in mosquito ecology, in particular their method of surviving the dry season and their dispersal rates.

**Conclusions:**

The modelling presented here indicates that considerable suppression of vector populations can be achieved within a few years of using a female sterility gene drive, though the impact is likely to be heterogeneous in space and time.

## Introduction

There is a great need to develop new tools to complement current measures to combat the burden of malaria in countries where it is endemic [1, 2]. One promising approach is to use a gene drive to suppress vector populations or to alter their capacity to transmit malaria. Gene drive systems based on natural or artificial homing endonucleases can in principle spread rapidly through populations, even if they reduce individual fitness [3–6].

Modelling studies have shown that female fertility traits are an attractive target for gene drive systems designed to bring about population suppression [7–9]. Several CRISPR-Cas9 driving transgenes have now been developed that target genes essential for female fertility in *Anopheles gambiae* mosquitoes, the major malaria vector in Africa, including three whose inheritance has been studied in cage experiments (one that targets the AGAP007280 gene [10] and two that target the doublesex gene (*dsx*; AGAP004050) [11, 12]). In population cage experiments, the frequency of the transgene that targeted the AGAP007280 gene initially increased, causing a reproductive load, yet then declined as fully fit alleles resistant to the drive emerged and were selected for [13].

The second transgene target (*dsx*) is far less susceptible to the emergence of resistance: the target site is in a region that is highly conserved among anopheline mosquitoes and hence sequence variants are likely to be non-functional [11]. In population cage experiments, chromosome repair did create alleles that were resistant to further homing, yet unlike the first transgene these alleles did not restore female fertility [11, 12]. In the absence of any functional *dsx* gene product, females are sterile because they develop a phenotype with both male and female characteristics, lacking functional ovaries and spermathecae; they are also unable to take a blood meal or produce any eggs [11]. The transgene developed by Simoni et al. [12] also encodes an endonuclease, I-PpoI, which is expressed during male meiosis and which cuts ribosomal DNA located on the X chromosome. This component thus induces male bias in the progeny of male carriers, which may hasten suppression [12].

Females with a single copy of the *dsx*-targeting transgene had reduced fertility compared to wildtype females, and two not mutually exclusive explanations have been suggested for this [11, 12]. First, there may be a somatic expression of the nuclease which interferes with female development. Second, there may be parental deposition of the Cas9 nuclease in the sperm or egg, so that females with a transgenic parent may suffer reduced fertility even when they do not themselves carry the transgene. Both possible effects are expected to reduce but not stop the drive of the transgene in susceptible populations [9]. In each study, the frequency of the *dsx*-targeting transgene increased to fixation resulting in population elimination, after 8–12 generations starting from 12.5% frequency [11] and after 10–14 generations starting from 2.5% frequency [12].

The encouraging results of [11, 12] suggest *dsx*-targeting transgenes may be candidates for deployment in the field. To assist in their evaluation, we here model the spread of a *dsx*-targeting gene drive in *An. gambiae* populations in a one million-square kilometre area of West Africa centred on Burkina Faso. We consider both ‘standard’ *dsx*-targeting transgenes, which affect female fertility in the manner of [11], and transgenes that additionally induce a paternal male bias in the manner of [12]. The model assumes that mosquito populations are located at human settlements (of which there are over 40,000 in the region) and that vector population dynamics are influenced by local seasonal rainfall patterns and the presence of nearby rivers and lakes. We compare the outputs of the spatial model with a much simpler non-spatial model that is analytically tractable.

In a previous study, we used the underlying spatial malaria vector model to explore the deployment of a different driving genetic construct involving an endonuclease on the Y chromosome which cuts the X chromosome so that the progeny of carrier males are all sons causing a male-biased sex ratio [14]. Non-spatial models predict that such a driving Y chromosome will rise to fixation in populations where it is introduced, causing population suppression or extinction [7]. In the West African spatial model, we found that a polymorphism often occurred where the wild type persisted through colonisation-extinction dynamics. Populations are locally extinguished by the driving Y chromosome only for the vacated sites to be recolonised by mosquitoes dispersing from unaffected populations carrying normal Y chromosomes [8, 14–16]. Population suppression still happened with this form of dynamics, but it was not as great as in the model without spatial structure.

The nature of the colonisation-extinction dynamics observed in the spatial model was influenced by the local climate and environment. In regions with strong seasonal fluctuations in mosquito numbers, populations that harboured the driving Y chromosome were at risk of extinction during the dry season even if the driving Y had not reached fixation. This accelerated the rates of colonisation and extinction and resulted in a reduction of the average extent of suppression. The local dynamics were also affected by the density of human settlements in a region; cycles of colonisation and extinction tended to be slower and more irregular in sparse compared with crowded regions due to less frequent movements of mosquitoes between sites. We found sustained suppression or extinction was possible in regions with year-round mosquito breeding habitat and a high density of population sites. There was thus a heterogeneous impact of the driving Y chromosome on mosquito density across the region we modelled.

A transgene targeting the *dsx* gene has different dynamics and is more complex to model than a driving Y chromosome because the construct is transmitted through females as well as males and also due to the complications of non-functional resistance alleles and parental effects. We explore how these factors interact with climatic and environmental variables to determine population suppression in an area of high malaria prevalence that may be considered for genetic vector control.

## Results

### Population suppression across the study area by an ‘ideal’ drive allele

We first consider a drive allele that is ‘ideal’, in the sense that its fitness effects are completely recessive and it causes no parental effects on its offspring (i.e. heterozygous females are always fully fertile). In line with experimental data, we assume high but not complete homing (95% in both males and females [11]) and that half of non-homed alleles form non-functional resistance alleles ( [17]; henceforth ‘r2 alleles’). We suppose the drive allele has no effect on the sex ratio of offspring, though below we will consider transgenes that induce male-biased sex ratios in addition to their effects on female fertility. Absent from this model is the potential emergence of a resistant allele that restores *dsx* function (an ‘r1 allele’). We assume such alleles will not emerge in the case of *dsx*-targeting transgenes due to the highly conserved sequence of the *dsx* gene [11], though the potential impact of very rare r1 alleles will be addressed in the ‘Discussion’ section.

The non-spatial model predicts such a drive allele will rise to 96.2% from a small initial introduction with the remaining percentage made up of wildtype alleles (1.3%) and r2 alleles (2.5%) which persist in a mutation-selection balance. Such a predominance of non-functional alleles is predicted to result in a genetic load of 0.974, leading to population extinction given our assumed population growth rate when rare of 18.9 (see the ‘Methods’ section).

**Fig. 1.**
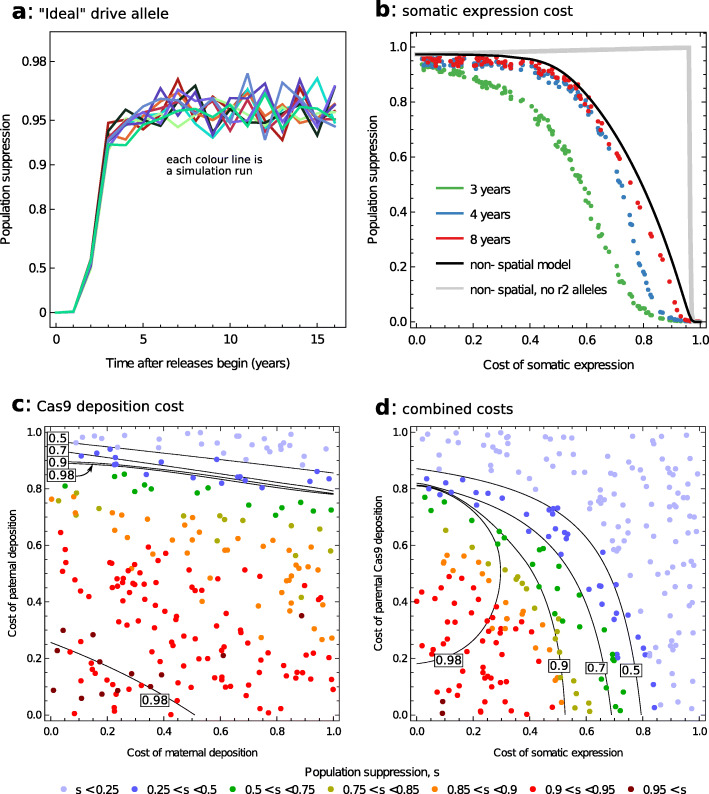
Predicted population suppression depends on the costs of the drive allele to the fertility of heterozygous females. The ideal scenario is that the drive allele is fully recessive and so heterozygous females are fully fit (**a**), though some degree of dominance may arise from somatic expression of the drive allele (**b**, **d**), and parental effects caused by deposition of Cas9 in sperm or egg may also reduce heterozygous fitness (**c**, **d**). Dots in **c** and **d** indicate the 8-year suppression predicted by the spatial model. All results shown follow the same default release strategy described in the text, of 5000 males released in 1% of human settlements per year, which are selected at random independently each year

We simulated the addition of heterozygous adult male mosquitoes each year to sites chosen at random and calculated the population suppression as the reduction in the number of biting female mosquitoes relative to a non-intervention baseline (see the ‘Methods’ section). The number of release sites per year is important in determining how quickly widespread suppression is achieved, while not affecting the eventual suppression level (Additional file 1: Fig. S1). The number of mosquitoes liberated per release, by contrast, makes little difference to either short- or long-term suppression. Significant suppression can be achieved within 4 years if there are at least a few hundred release sites per year. If releases of 5000 males are made at 1% (434) of sites each year, the female population is reduced by ~ 95% after about 4 years (~ 95% suppression; Fig. 1a; 4-year suppression ranged from 92.6% to 95.8% with mean 94.6% from 10 simulations). In the first few years after releases begin, somewhat greater suppression is achieved if the release sites are distributed on a regular grid rather than chosen at random (this resulted in 95.5% (94.5–97.2%) suppression). This difference diminishes with time, however, and is not apparent after 8 years.

Note that females that lack a functional *dsx* gene are ignored when we calculate suppression because they are unable to take blood meals. In the above example with randomly located release sites, the inclusion of *dsx*-negative females would lower the population suppression predicted by the spatial model to ~ 92%. We discuss below the differences in suppression predicted by the spatial and non-spatial models.

### Costly drive alleles

#### Somatic expression of Cas9

Next, consider the possibility that there is some somatic expression of the Cas9 nuclease encoded by the drive allele, yet there is no parental deposition of the protein. We assume the somatic expression of Cas9 reduces the fertility of drive heterozygous females because it partly or completely prevents the production of the *dsx* gene product in cells that require it. The non-spatial model predicts that such a drive allele will cause population extinction provided the reduction in heterozygous fertility is less than ~ 0.44. For costs greater than this, suppression is predicted to decline to a limit of 0 if heterozygous females are completely infertile (black line in Fig. 1b).

Somatic expression reduces the impact of the drive for two reasons. First, there is a direct cost to the transmission of the drive allele from one generation to the next, because drive heterozygous females will have fewer offspring. Second, the cost increases the relative fitness of r2 alleles over the drive allele, because r2/wildtype heterozygous females are assumed to be fully fertile, and so the frequency of r2 alleles increases at the expense of drive alleles. We find the latter cost has a greater effect on the level of predicted suppression; if no r2 alleles are produced, population extinction is predicted by the non-spatial model for costs less than ~ 0.96, a higher threshold (compare the grey and black lines in Fig. 1b). Both factors act to slow the spread of the drive allele, however, so that as rates of somatic expression increase so does the time it takes to reach maximum population suppression.

The spatial model predicts a similar response to somatic Cas9 expression, though long-term suppression is consistently a few percentage points lower than the genetic load predicted by the non-spatial model (Fig. 1b). Stable levels of suppression are attained after about 4 years when somatic expression costs are 0.4 or less, while for higher costs, the time to equilibrium requires a few more years.

#### Parental deposition of Cas9

Now consider the possibility that *Cas9* enters the zygote through deposition in the gametes so that female fertility is reduced when either the mother or the inseminating father carries the drive allele (with the paternal and maternal effects possibly being different). We assume that there are no additional genotype-specific costs, so that drive heterozygous females have wildtype fitness. Analysis of the non-spatial model suggests that deposition costs can prevent the transgene bringing about population extinction, though only if the effects of paternal deposition are relatively strong (Fig. 1c). Thus, in the absence of maternal effects, paternal effects must cause a greater than ~ 90% reduction in fitness to stop extinction, and as maternal effect costs rise to 100%, this threshold drops to ~ 80%. The greater importance of paternal over maternal deposition is because both drive homozygous and heterozygous males are assumed to be fully fertile, so individuals are more likely to have a father than a mother with the drive allele (see also [9]). Parental effects have less impact than somatic expression, because they affect females with r2 alleles as well as females with drive alleles.

As parental deposition costs increase from zero, there is a small increase in population suppression (genetic load) due to reduced female fecundity (Additional file 1, Fig. S2). However, for higher costs, there is an abrupt transition in dynamics with much reduced genetic load and hence the absence of population extinction. For the case of equal paternal and maternal costs, this threshold occurs when costs exceed 0.813 (Additional file 1, Fig. S2). Mathematically, the system displays two equilibria, with low and high genetic loads. In the absence of, or with moderate, deposition costs, the high and low genetic load equilibria are respectively stable and unstable, but this switches at the threshold giving the abrupt transition. Biologically, below the threshold, the selective advantage that drive alleles have over wildtype alleles due to homing is sufficient to outweigh the costs of parental deposition, but above the threshold, the balance tips the other way allowing the wildtype and r2 alleles to invade. Note that high deposition costs can have a large effect in slowing down the approach to equilibrium (Additional file 1: Fig. S3).

Parental deposition has a greater effect in reducing population suppression in the spatial compared to the non-spatial model, though again paternal deposition costs are more important than maternal. Thus, to achieve at least 90% suppression after 8 years of releases (roughly equivalent to population extinction in the non-spatial context), the reduction in fitness due to paternal deposition must be less than 60% (no cost of maternal deposition) to 40% (maternal deposition renders females infertile). We hypothesise that the dynamics of the spatial model are also affected by underlying alternative stable and unstable equilibria whose precise properties are influenced by local environmental factors in a way that lowers the threshold of the transition to the low genetic load equilibrium. As in the non-spatial model, we find deposition costs slow the effect of the transgene in achieving population suppression.

#### Combined somatic expression and parental costs

We also consider the possibility of both somatic expression and parental Cas9 deposition acting in concert, assuming the costs of the latter to be the same for maternal and paternal depositions (Fig. 1d). The non-spatial model predicts both types of cost act to reduce eventual suppression though, as discussed above, the somatic costs have a somewhat greater effect. The spatial model, by contrast, predicts that the cost of Cas9 deposition is approximately equal to the cost of somatic expression. Again, we hypothesis that the differences are due to subtle interactions between the genetic dynamics and spatially heterogeneous environmental factors.

### Paternal male bias

**Fig. 2.**
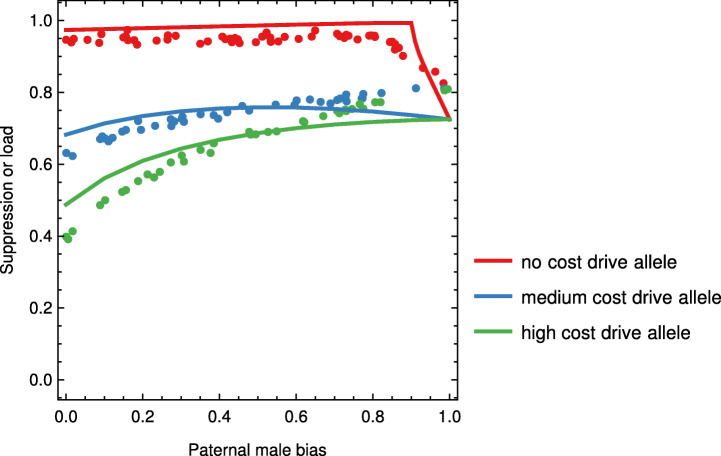
The effect of engineered paternal male bias on the performance of the drive allele, based on the non-spatial (lines) and spatial (dots) models. The three drive alleles differ in somatic expression, so that heterozygous females have a fertility cost of 0 (‘no cost’), 0.7 (‘medium cost’), and 0.8 ‘high cost’ (chosen to result in three equally spaced levels of population suppression in the absence of paternal male bias, cf. Figure 1b; parental costs are not considered). For the spatial model, population suppression is computed after 8 years of releases following a release strategy as defined in Fig. 1

We next suppose a paternally expressed male-biasing component has been added to the transgene, in line with the construct developed by Simoni et al. [12]. We varied the extent of male bias from 0 to 1 (when all progeny of transgenic males are male), for three levels of fitness cost to the heterozygous females (Fig. 2). The non-spatial and spatial models both indicate that paternal male bias will be of little benefit to a strong drive allele. Indeed, high levels of male bias reduce the potential of the transgene to suppress populations because this results in the creation of fewer transgene homozygous females (see also [12]). However, the suppression potential of moderate and weak transgenes can be substantially enhanced by paternal male bias. This is because the presence of transgenic males skews the population sex ratio towards males, thus reducing both the number of adult females and the population growth rate. In the limit of paternal bias equalling one, there is no difference between drive alleles that differ in heterozygous female fitness cost, because heterozygous females are not produced in this scenario.

### Spatial variation

**Fig. 3.**
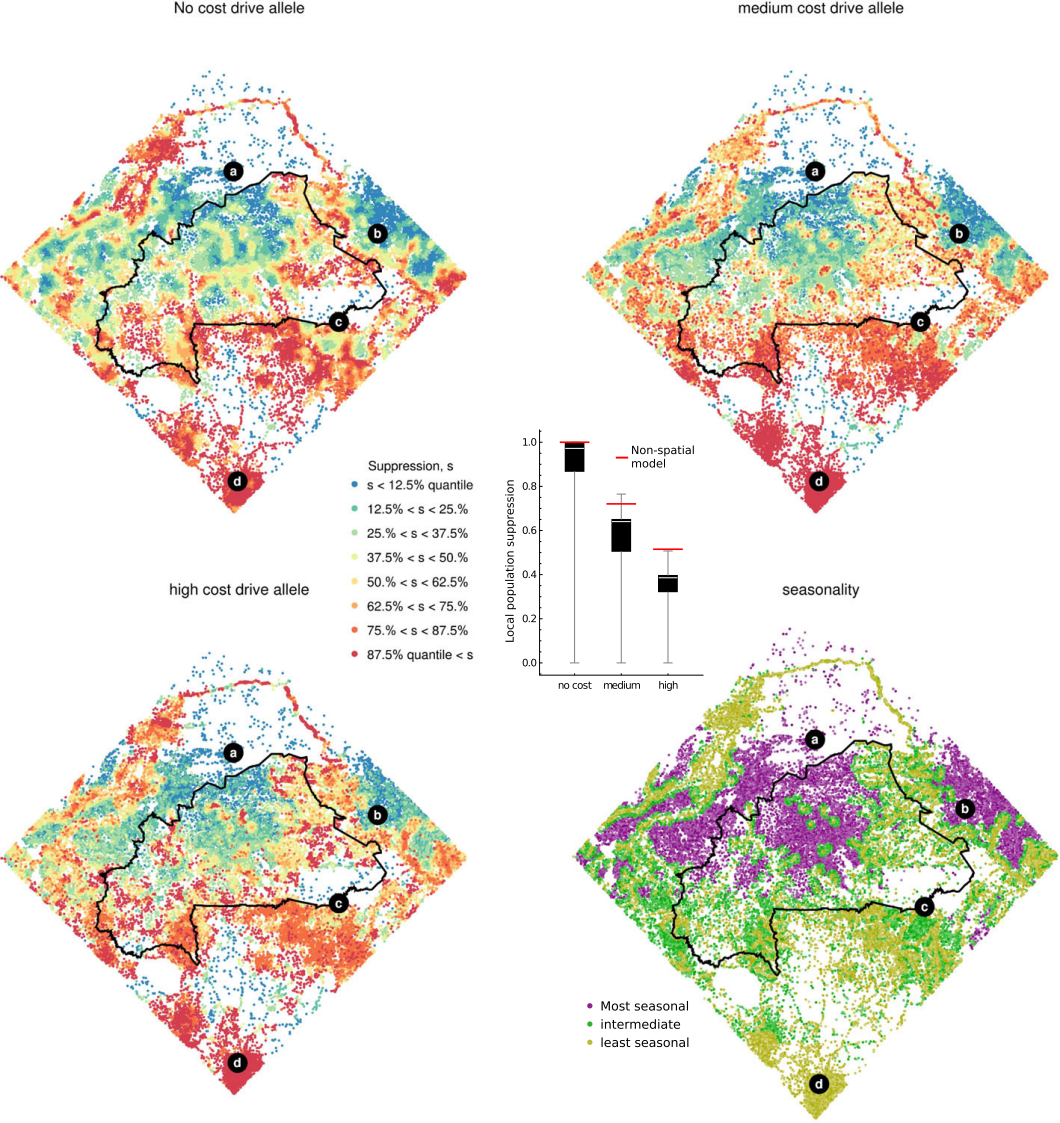
Spatial variation in suppression across the study area, depending on the heterozygous female fertility costs. As Fig. 2, the three drive alleles differ in somatic expression costs (0, ‘no cost’; 0.7, ‘medium cost’; 0.8, ‘high cost’), while both parental costs and paternal male bias are set to 0. For each suppression plot (**a**, **b**, **c**), the quantiles are computed from the set of site by site suppression levels after 8 years of releases, averaged from ten simulation runs. The release strategy is as defined in Fig. 1. The corresponding seasonality quantiles (**d**) are computed from the yearly minimum population sizes in the absence of drive alleles, with most and least seasonal sites defined as the tertiles with the lowest and highest yearly minimums, respectively

**Fig. 4.**
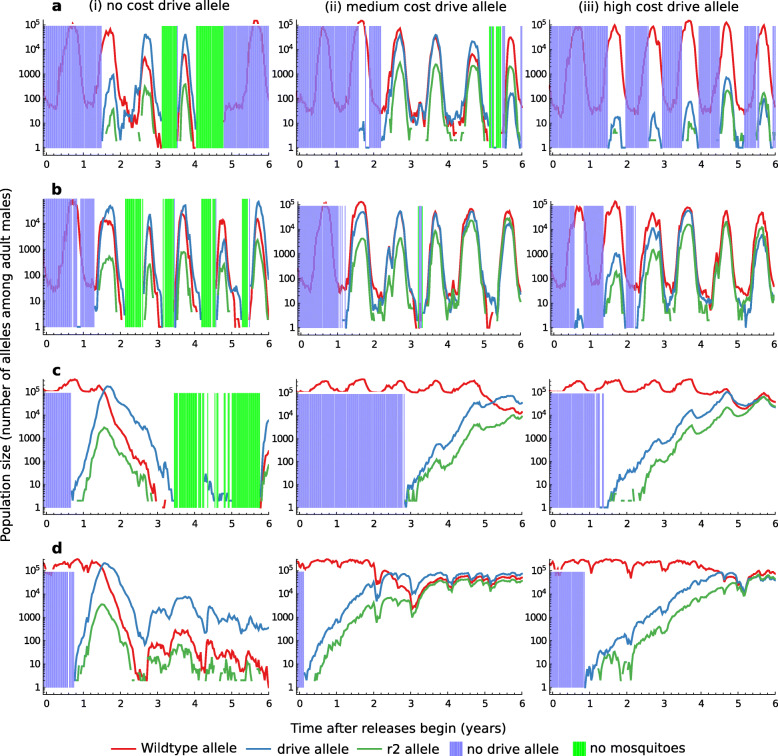
Examples of local population dynamics at four sites shown in Fig. 3. Drive alleles are as defined in Fig. 3, and the release strategy is as defined in Fig. 1

To understand why the spatial models consistently predict lower population suppression than the non-spatial models, we next consider the geographic variation in suppression for three constructs that again differ in their fitness costs experienced by heterozygous females (Fig. 3; assuming no paternal male bias). Though these alleles differ considerably in average suppression after 8 years (from 38.5% for the weak allele to 95.8% for the strong), they share a remarkably similar pattern of the strongest suppression in the least seasonal regions. However, the apparently consistent role of seasonality masks a number of qualitative differences in how strong and weak drive alleles influence local population dynamics. To illustrate these differences, we study four exemplar sites, shown as points a–d in Fig. 3. Site ‘a’, in Mali, is highly seasonal and also somewhat isolated from other settlements; site ‘b’, in Niger, is highly seasonal and highly connected; site ‘c’, on the Benin-Burkina Faso border, is somewhat isolated yet due to its proximity to the Pendjali river is assumed to have year-round larval habitats; and finally, site ‘d’, which is in Ghana, is both highly connected and assumed to have year-round larval habitat due to a mild dry season in this region. Figure 4 shows the typical simulated dynamics at each site and for each of the three drive alleles described above.

If the drive allele is strongly suppressing (left column of Fig. 4), colonisation-extinction dynamics occur in much of the study area (and are seen in all example sites except site ‘d’). The colonisation and extinction cycles are fastest in sites with strongly seasonal environments (sites ‘a’ and ‘b’) and most regular in sites within locally dense networks of human populations (site ‘b’). In these densely populated regions, frequent dispersal of mosquitoes between sites ensures their dynamics are correlated and similar levels of suppression are observed in all locations. In more sparsely populated regions, there is greater variation in dynamics across sites: populations are unaffected in some locations because the transgene has not established or has become extinct, whilst sites that have been successfully colonised by the transgene and become extinct may remain empty for several years. Suppression is therefore less variable in regions with a high density of human settlements (Additional file 1: Fig. S4). In regions that both lack a severe dry season and have a high human density, we find strong and continuous suppression is typical rather than colonisation-extinction dynamics (site ‘d’). The high human density facilitates immigration of mosquitoes from adjoining regions, which in this case is sufficient to maintain population persistence.

If the drive allele is moderately or weakly suppressing, populations rarely become extinct even in severely seasonal populations (middle and right columns of Fig. 4). In highly seasonal sites, however, populations may become so small in the dry seasons that allele frequencies are strongly affected by genetic drift (sites ‘a’ and ‘b’). During these times, the drive allele is at risk of becoming either lost or reduced to a low frequency. Loss of the drive allele appears more common in remote than well-connected sites, since a remote population may receive only a small number of mosquitoes carrying the allele in any rainy season (compare the performance of a weak allele in sites ‘a’ and ‘b’). Whether or not the drive allele tends to survive the dry season, however, the consequences of ‘dry season drift’ is to disrupt the suppression effect of the drive allele and so to reduce its efficiency. In populations with mild seasonality, alleles with medium and low strength drive cause stable suppression, as predicted by the non-spatial model (sites ‘c’ and ‘d’).

### Robustness of the results

**Fig. 5.**
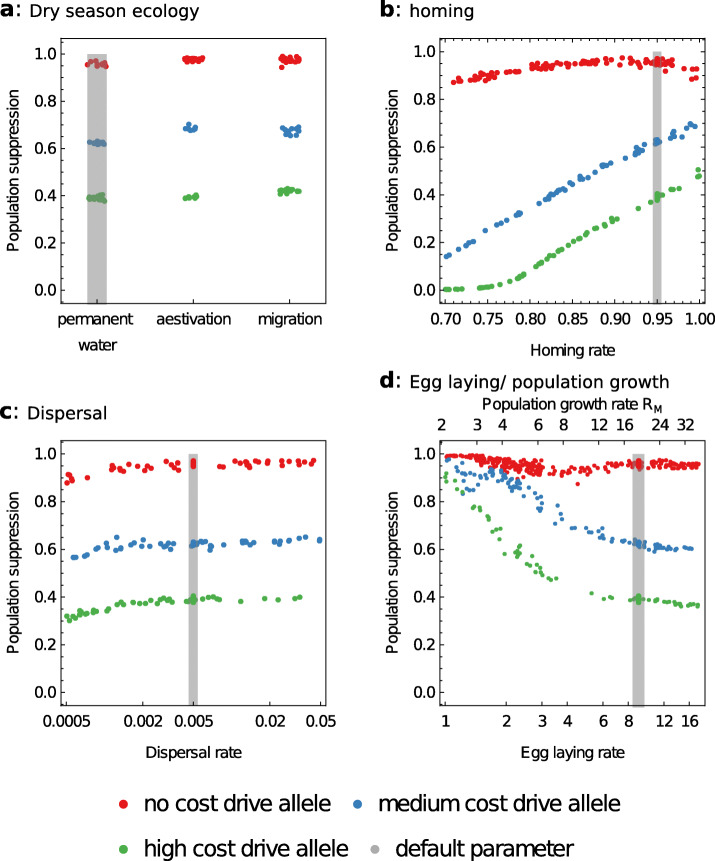
The robustness of our results to uncertainty in dry season ecology, homing, dispersal, and egg laying rate. Drive alleles are as defined in Fig. 3, and the release strategy is as defined in Fig. 1

The results presented so far are based on our best estimates of model parameters. To explore the robustness of our results to uncertainty in these parameters, we next investigate the importance of four factors: mosquito dry season ecology, homing rate, dispersal rate, and egg laying rate (Fig. 5).

#### Dry season ecology

So far, we have assumed all human settlements contain a small amount of permanent larval habitat in addition to habitat associated with rainfall or the proximity of rivers and other water bodies. This ensures that mosquito populations are maintained in nearly all sites in the absence of drive alleles, as observed in the field. However, it is very difficult to find larval sites in many areas during the dry season, so we now suppose that populations are instead maintained by either the dispersal of adult females using high-altitude winds or the aestivation of adult females. Following our previous studies using the same underlying model [14, 18], long-distance dispersal is modelled by assuming a fraction of adult female mosquitoes are redistributed by seasonal prevailing winds. Aestivation is modelled by assuming that a fraction of adult female mosquitoes are dormant each dry season, meaning their mortality risk is reduced but they do not lay eggs. For each process, we set parameters that ensured mosquitoes were present in most human settlements in the rainy season (on average > 99.4% sites).

The different assumptions about dry season ecology had only modest effects on the average level of population suppression after 8 years across the study area (Fig. 5a). The inclusion of aestivation and long-distance dispersal both resulted in somewhat higher suppression than assuming the presence of permanent water bodies in the case of the strong and medium strength drive allele, while long-distance dispersal resulted in somewhat higher suppression in the case of the weak drive allele (Additional file 2: Table S1). The small effect sizes are chiefly because the dry season assumptions are only important in regions where seasonality is strong. Long-distance dispersal makes little difference to the suppression caused by a strong drive allele, yet tends to increase the long-term suppression caused by medium and weak drive alleles in seasonal locations (Additional file 1: Fig. S5). This is because dispersal connects large year-round populations to sites with strong seasonality, and thus reduces the role of dry season stochasticity.

Aestivation tends to reduce the initial impact of medium and weak drive alleles in seasonal locations, because the inactivity of mosquitoes over the dry season slows the spread of the drive allele (Additional file 1: Fig. S6). This effect diminishes as the drive allele spreads throughout the study area, and in most locations, aestivation either increases or has no effect on suppression after 8 years. The precise effect of aestivation on highly seasonal locations depends on how it influences the fluctuations in population size in those locations. In moderately seasonal locations, aestivation increases suppression because it reduces the dry season bottlenecks in population size that promote stochasticity. In the most strongly seasonal regions, however, aestivation results in lower suppression even after 12 years (blue areas in Fig. S6). In these locations, our model predicts the pre-intervention populations are smaller if mosquitoes rely on aestivation rather than permanent water, and the overall extent of stochasticity is thus greater. These results are sensitive to the parameters we use to model dry season ecologies and so should be treated with caution, though they emphasise that the effects of dry season ecology may be quite subtle in the most strongly seasonal locations.

#### Homing rate

While it is relatively straightforward to measure homing rates in a laboratory (e.g. [10, 11, 19]), it is more difficult to determine how consistent homing frequencies will be throughout the range of genetic backgrounds a drive allele will potentially encounter. The relationship between the homing rate and the impact of a gene drive is also of interest to molecular biologists who may be able to manipulate this parameter.

The non-spatial model predicts that population suppression will increase with the homing rate, a relationship also shown by the spatial model with weak or medium-strength drives (Fig. 5b). A different pattern is seen for strong drive alleles, which over the range we model (0.7–1) are less sensitive to homing rate and show peak suppression at an intermediate value (0.9). The reduction in suppression at very high homing rates occurs because strong drive causes populations to become extinct more rapidly, which can give rise to extinction-colonisation dynamics in locations that would otherwise be permanently suppressed.

#### Dispersal

The best estimates of inter-village *Anopheles* dispersal come from three West African mark-release-recapture experiments using locally caught *An. Gambiae s.l.* mosquitoes, whose measurements imply dispersal rates of 0.005–0.034 inter-village movements per mosquito per day in our parameterisation [18]. We took the lower value of 0.005 to be our default dispersal rate because the experiments were conducted in closely neighbouring villages, yet the possibility that this is an order of magnitude too high or too low cannot be discounted on the basis of so few data. We find that the predicted suppression after 8 years is not sensitive to the assumed dispersal rate provided it is above a threshold of approximately 0.001 movements per mosquito per day (Fig. 5c). Suppression is reduced if dispersal is below this threshold, for example, by approximately 15% if the dispersal rate is 0.0005 movements per mosquito per day.

The dispersal rate plays a larger role in determining the time to reach equilibrium (Additional file 1: Fig. S7). Unsurprisingly, drive alleles take longer to have an impact across the study area for lower dispersal rates, though the size of this effect depends on the strength of the drive allele. For a given dispersal rate, strongly suppressing drive alleles spread faster than weakly suppressing alleles, and they are also less affected by uncertainty in the dispersal rate.

#### Population growth rate

Finally, we vary the egg laying rate of wildtype female mosquitoes, keeping the relative fecundities of different genotypes the same. This parameter is directly proportional to the intrinsic population growth rate, *R*_*m*_. Populations with low growth rates are expected to be more affected by suppression drive alleles, because they have less capacity to withstand reductions in fertility (e.g. [7]). This explains the clear negative relationship between growth rate and suppression seen for the medium and low strength drive alleles and for the strong drive allele if the growth rate is small (Fig. 5d). For higher growth rates (> 8), we see a modest positive relationship between growth rate and suppression for the strong allele. In the presence of a strong drive allele, a small growth rate hastens the extinction of local populations, which both increases suppression locally and also promotes colonisation and extinction dynamics. These counterbalancing factors give rise to the shallow trough-shape of the relationship.

## Discussion

Our simulations demonstrate the clear potential of driving endonuclease genes targeting female fertility to reduce malaria vector populations on a regional scale. We investigated how this potential is affected by the strength of the fertility reduction, variation in the environment through which the gene must spread, and different assumptions about uncertainties in mosquito ecology. For most of the parameter space, simple analytically tractable non-spatial models provide a good approximation of the dynamics of the more complex model that includes the spatial distribution of adult and larval mosquito resources. These models are thus very useful for rapidly assessing the differences among gene drive scenarios. However, our results also demonstrate that more detailed spatial models are needed to explore subtle interactions between local environmental factors and the impact of these kinds of drive alleles.

The spatial model has also enabled us to address factors that are beyond the scope of non-spatial modelling, such as the rate of spread of a drive allele in different parts of a landscape and the time required for suppression to occur. Though we have not done so here, detailed models are also very valuable in designing deployment strategies (e.g. [20, 21]).

We found population suppression to be reduced in regions that undergo large seasonal fluctuations in vector densities, and this appears to be the principle reason that our non-spatial model tended to predict somewhat lower population densities than the full spatial model. We found a similar result in our studies of the spread of a driving Y chromosomes over the same region of Africa [14], suggesting that the effects of seasonality described here may be quite general to gene drive constructs that are designed to cause population suppression. Seasonality reduces suppression because dry season conditions promote genetic drift which is disruptive to gene drive, sometimes causing the drive allele to become locally extinct.

The importance of seasonality will depend on the dry season ecology of the mosquito vectors being targeted. Most of the simulations presented here assumed there were some small permanent water bodies in all settlements, which ensured at least a few mosquitoes survived each dry season at each site. It seems likely that such water bodies will exist in some but not all locations, yet even where they do it is difficult to know whether the very small dry season population sizes predicted by our model are realistic. There is mounting evidence that some anopheline mosquitoes survive through the dry season by aestivating in unknown shelters [22–28], while others are transported large distances by high-altitude winds [23, 29]. However, it remains unclear how significant these processes are to the widespread maintenance of populations in highly seasonal regions. Our simulations of the two processes revealed that both may reduce the role of seasonality on the impact of a drive allele in some regions, though aestivation had the opposite effect in other regions. Our results also suggest that widespread aestivation will slow the initial spread of a drive allele, as we found previously for the case of driving Y chromosomes [14]. Further research on all aspects of anopheline dry season ecology will clearly be valuable in improving our ability to understand and predict gene drive in mosquitoes.

This study is motivated by the transgenic constructs developed by Kyrou et al. [11] and Simoni et al. [12], though we have considered a wider range of parameter values to reflect uncertainty in the precise behaviour of the construct under field conditions and to guide further developments. The strongest suppression is achieved by fully recessive drive alleles, yet our results suggest that substantial suppression can still be achieved by drive alleles that incur moderate heterozygous costs. Kyrou et al. found the fertility of heterozygous females produced by transgenic fathers and mothers to be respectively 78% and 35% less than wildtype females [11]. With these costs, our spatial model predicts population suppression after 8 years in the range of 64–69%. The construct developed by Simoni et al. was found to confer 93% paternal male bias, while the fitness of heterozygous females was 35% less than wildtype females [12]. With these parameters, our model predicts an 8-year suppression of 89–91%.

These levels of vector population suppression will clearly reduce disease transmission, though quantifying the extent is beyond the scope of this study. Other genetic constructs that may contribute to disease reduction include driving genes that express anti-*Plasmodium* phenotypes (e.g. [30]). It would be interesting to explore the possibility of combining such transgenes with constructs designed for suppressing populations, to see if they would achieve larger impacts than either would alone. This may be a particularly useful strategy in the case of moderate suppression constructs.

There are a number of ways this work could be extended to gain deeper insights into how driving endonuclease genes would affect mosquito populations. First, additional aspects of mosquito ecology could be incorporated. These include incorporating population spatial structure at a finer resolution than we have done here [8, 16], different forms of density dependence [31], and the influence of local ecology and topology on mosquito dispersal (e.g. [32]).

Second, we have not considered the possibility of resistance evolving to hinder the spread of a drive allele. The most obvious way this might happen is by the emergence of a resistant allele that restores dsx function (an ‘r1 allele’). Models of panmictic populations predict that functional resistant alleles will evolve quickly if they are occasionally created by the homing reaction of a drive allele, or if they pre-exist in the standing genetic variation [7, 33, 34], and lab studies have confirmed this dynamic [13, 35, 36]. Designing and engineering a genetic construct where resistant alleles are non-functional is the primary strategy to avoid this outcome and a major part of the reason for the interest in the dsx locus. Initial exploration using our non-spatial model provides some insights into the speed with which a pre-existing resistant allele will spread (Additional file 1: Fig. S8). We assumed r1 alleles with the same fitness as the wildtype are present in the population at an arbitrarily low frequency of 10^−8^ at the start of the simulation (we estimate there are approximately 10^9^ − 10^10^ mosquitoes in our study area). We found that while the drive allele initially spreads causing population suppression, the r1 allele subsequently increases in frequency excluding the transgene and restoring the population to its original density. This occurs more rapidly for strongly rather than weakly suppressing drive alleles, because the former reaches a high frequency more rapidly and it is then that the r1 allele becomes strongly selected. Weakly suppressing drive alleles, on the other hand, are likely to become lost from the population before the r1 allele reaches fixation, resulting in a population comprising both r1 and wildtype alleles. Depending on the detailed assumptions, the drive allele may cause substantial population suppression, and in a stochastic context possible local extinctions, before it is outcompeted by the r1 allele. The release of sequential drives, each at risk of resistance, is one strategy to counter resistance though avoiding it arising in the first place is always better. Means of achieving the latter include targeting conserved, functionally constrained parts of the genome and multiplexing, as previously proposed [34, 37, 38]. The target site of the dsx transgene developed by Kyrou et al. [11] is highly conserved among mosquito species, suggesting that functional escape mutants are likely to arise very slowly if at all. The additional male-biasing component of the transgene developed by Simoni et al. [12] is likely to further reduce the evolution of resistance, since resistant mutations are not selected in males. However, the size of the vector population across and beyond our study area is so large that resistance may arise at some time, and it will be important to model the consequences of this.

It is also possible that mosquito behaviours will evolve in response to the gene drive [31]. For instance, a recent modelling study showed that a propensity for sib-mating might evolve in response to a suppression gene drive [39]. It is unclear whether this particular behaviour could evolve quickly enough in anopheline mosquitoes to be important.

Finally, the geographical domain of the study could be extended to other parts of West Africa and beyond where there are different spatial patterns of adult and larval mosquito resources. This would be computationally demanding and might require parallel computing methods or the development of appropriate approximations.

## Conclusion

Population modelling has an important role in discussions of whether and how gene drive technology should be used in the field. In this study, we have explored releases of a driving endonuclease gene targeting female fertility for mosquito vectors of malaria in an area of West Africa large enough to exhibit considerable environmental and social heterogeneity. We have shown that repeated introductions of these modified mosquitoes could be an effective means of reducing vector numbers at a regional scale.

## Methods

The two models we use in this study, the non-spatial model of population genetics and the individual based spatial simulation model, make the same assumptions with respect to the genetic architecture. We therefore first describe the simpler non-spatial variant before describing how the spatial model implements the genetic assumptions. The non-spatial model is written in the Mathematica language (Wolfram Research), while the spatial model is encoded in C++; the codes for both are available from Github (https://github.com/AceRNorth/BurkinaMosquitoModel).

### Non-spatial model

The model considers three types of allele competing at a locus: the wildtype allele (the *doublesex* gene), the homing allele, and a non-functional mutant allele that is resistant to homing. Homing occurs in wildtype/homing heterozygotes of both sexes, leading to a biased transmission of the homing allele from one generation to the next. The chromosomal cleavage caused by Cas9 which is a component of homing may also create non-functional resistant alleles, from the processes of non-homologous end joining (NHEJ) and microhomology-mediated end joining (MMEJ) [11, 13, 35] or from incomplete homing [35]. Females are completely sterile if they lack at least one copy of the wildtype allele, while wildtype/homing heterozygous females may have reduced fertility to allow the possibility of somatic expression of Cas9. The model also encodes the possibility of parental effects of the homing allele and paternal male bias. The equations of the model are given in the supplementary material (Additional file 3).

#### Non-spatial population suppression

We define ‘genetic load’ by the fertility of the female population at equilibrium in comparison with a pure wildtype population. Specifically, $L=1-{\sum}_{i\in G}{f}_i{F}_i^{\ast }$ where *G* is the set of all female genotypes, *f*_*i*_ is the fertility of genotype *i* (relative to the homozygous wildtype genotype), and ${F}_i^{\ast }$ is the frequency of genotype *i* at equilibrium. The genotype frequencies at equilibrium are determined from a numerical iteration of the model. At this equilibrium, the growth rate of the population, ${R}_m^{\prime }$, is the growth rate of the pure wildtype population, *R*_*m*_, multiplied by the load reduction, 1 − *L*. The critical load required to drive a population extinct, *L*_*c*_, is the load for which ${R}_m^{\prime }=1$, giving *L*_*c*_ = 1 − 1/*R*_*m*_. We determine the growth rate by computing the lifetime production of female offspring using the parameters of the spatial model in the case of no density dependence.

Specifically,

$$ {R}_m=\left(\mathrm{juvenile}\ \mathrm{survival}\ \mathrm{probability}\right)\times \left(\mathrm{survival}\ \mathrm{for}\ \mathrm{one}\ \mathrm{day}\ \mathrm{to}\ \mathrm{become}\ \mathrm{mated}\  female\right)\times \left(\mathrm{eggs}\ \mathrm{per}\ \mathrm{female}\ \mathrm{per}\ \mathrm{day}\right)\times \left(\mathrm{adult}\ \mathrm{female}\ \mathrm{life}\ \mathrm{expectancy}\right)\times \left(\frac{1}{2}\right) $$

$$ ={0.95}^{10}\times 0.875\times 9\times 8\times 0.5\approx 18.9. $$

The critical load is thus *L*_*c*_ = 0.947.

### Spatial model

The underlying spatial simulation model has been described previously [14, 18], and we thus give only an overview here and refer the reader to [18] for full details. The overarching population is sub-divided into a network of randomly mixing mosquito populations, each of which is located at the site of a human settlement. Within each population, mosquitoes are classified by life stage (juvenile or adult), sex, and genotype. Adult females are further classified by whether or not either parent had a homing allele, and whether or not they have mated; if they have, the genotype of their mate is also recorded. Life history processes (survival, mating, egg laying) are stochastic, though we assume larval development takes a fixed 10 days (from egg to eclosion) if they survive this long. Larval survival rates decrease with the number of larvae in a population, to an extent that depends on local rainfall and local groundwater associated to rivers and lake edges. This ensures that, in the absence of the drive allele, each population tends to a carrying capacity that is constantly shifting in response to rainfall and location. Each time an adult female lays eggs, the zygotic genotypes are each randomised depending on her and her mate’s genotype and assuming the same inheritance architecture as the non-spatial model. We assume a fraction of mosquitoes disperse each day, with the destination site selected with a probability that decreases with distance from among all the sites that are within 12 km from the focal site.

The model takes as inputs the locations of human settlements (42,360 in the study area) collected by the United Nations Office for the Coordination of Human Affairs [40], inland water data extracted from the digital chart of the world [41], and rainfall data from the ‘ERA-interim reanalysis’ (available from the European Centre from Medium-Range Weather Forecasts [42]). The default model parameters are the same as those used previously [14], except for the parameters of the gene drive constructs which are given in the text.

#### Spatial model population suppression

Population suppression in this model is defined as the reduction in the number of phenotypic adult female mosquitoes across the entire study area. At any time after the transgene has been released, the total number of phenotypic females is computed as the sum of all adult females except those that are either drive allele homozygous or drive allele/r2 allele heterozygous. To compute population suppression on a given date, the total number of phenotypic females on that date is divided by a reference for the corresponding total number of adult wildtype females in a simulation run where no drive alleles were released.

## Supplementary information

**Additional file 1: Figures S1-S8. Fig. S1.** Simulated population suppression from the spatial model after four and eight years of releases of the “ideal” drive allele, depending on the number of release sites per year and the size of the releases. **Fig. S2.** The non-spatial model shows a high degree of sensitivity to high Cas9 deposition costs, due to alternative equilibria being attractive depending on the precise parameters. In both panels, both and maternal parameters are equal, and we assume there are no somatic expression costs. **Fig. S3.** If the Cas9 deposition costs are close to, yet below, the threshold for converging to a high drive allele equilibrium (cf. Fig. S2), the genetic load on the population will also be high at equilibrium. However, the convergence to equilibrium is faster if these costs are not present (dashed lines). **Fig S4.** The joint influence of connectivity and seasonality on average population suppression. Connectivity of a site is defined as the number of neighbouring sites within a radius of 12km, and the three plotted connectivity levels are the tertiles of this measure across the study area. **Fig. S5.** Showing how the predictions change if mosquito populations are maintained by frequent long-distance migration rather than by small bodies of permanent water, which was the default assumption. (Figure 3. in the paper plots the spatial variance in suppression eight years after releases begin for the default case, thus corresponding to the middle row here). **Fig. S6.** As Fig. S5, but now assuming mosquito populations are maintained by adult female aestivation. **Fig. S7.** The transient dynamics of suppression for the three strengths of drive allele (cf. Fig. 2) and for three rates of dispersal. **Fig. S8.** Allele frequency dynamics predicted by a version of the non-spatial model that includes fully functional r1 alleles as well as non-functional r2 alleles discussed elsewhere in the text. The three drive alleles differ in somatic expression costs (cf. fig. 2). Note the different x-axes ranges among the three plots.

**Additional file 2: Table S1.** The mean, minimum, and maximum predictions of population suppression depending on the dry season ecology and the drive allele strength. Each result is from ten simulation runs.

**Additional file 3.** Equations of the population genetics that define the non-spatial model and underlie the spatial model.

## Data Availability

Settlement data collected by the United Nations Office for the Coordination of Human Affairs (OCHA [40];), inland water data extracted from the digital chart of the world (DCW [41];), and rainfall data from the ‘ERA-interim re-analysis’ (available from the European Centre for Medium-Range Weather Forecasts [42]).
