# Supplementary figures and images for: Modelling the suppression of a malaria vector using a CRISPR-Cas9 gene drive to reduce female fertility
Source: BMC Biol. 2020 Aug 11;18:98. doi: 10.1186/s12915-020-00834-z (PMC7422583; doi:10.1186/s12915-020-00834-z)

Fig. S1.

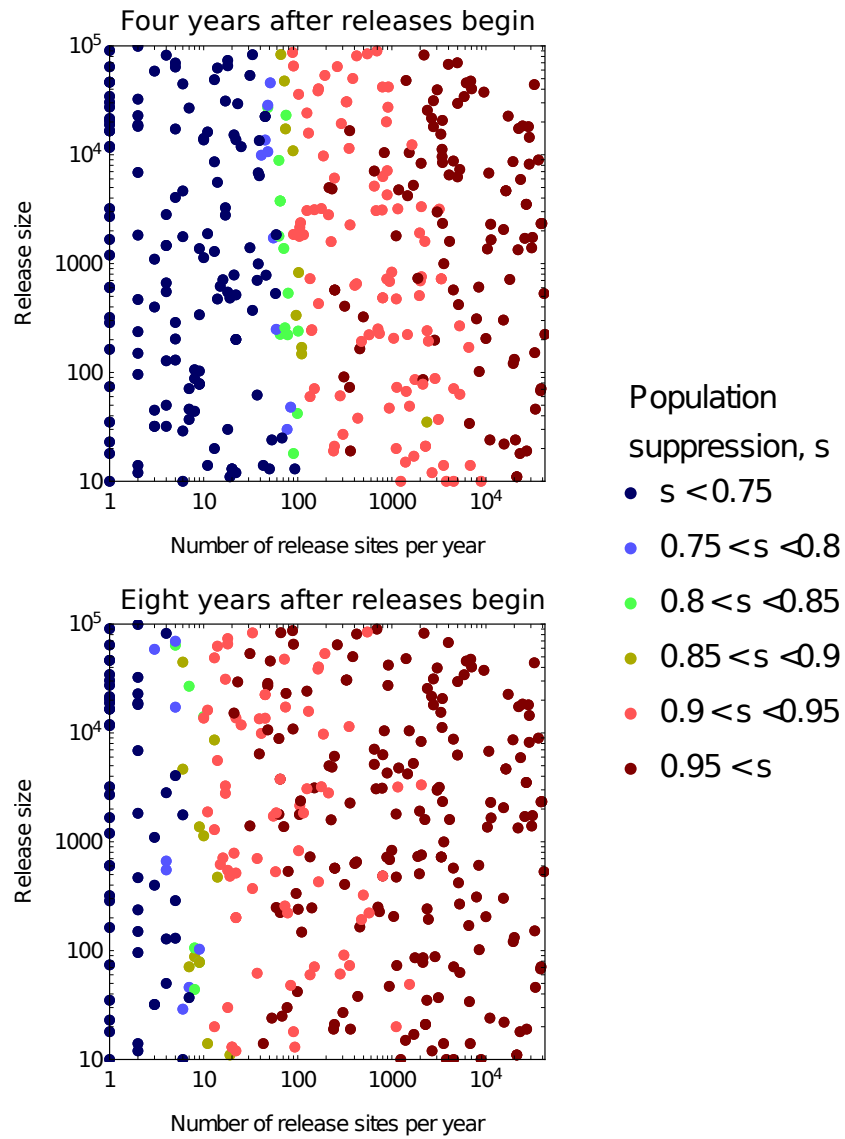

Fig. S2.

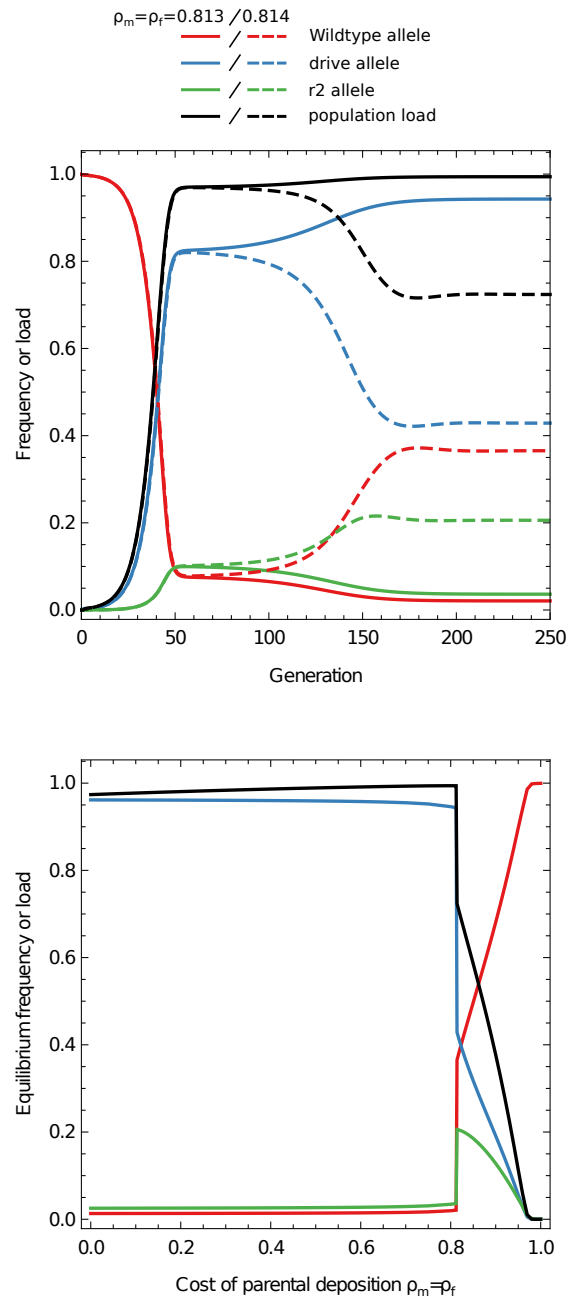

**Fig. S3.**

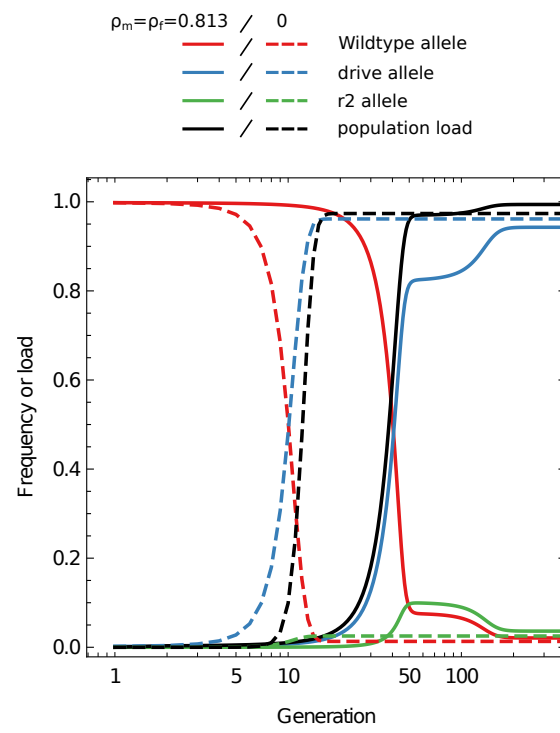

Fig. S4.

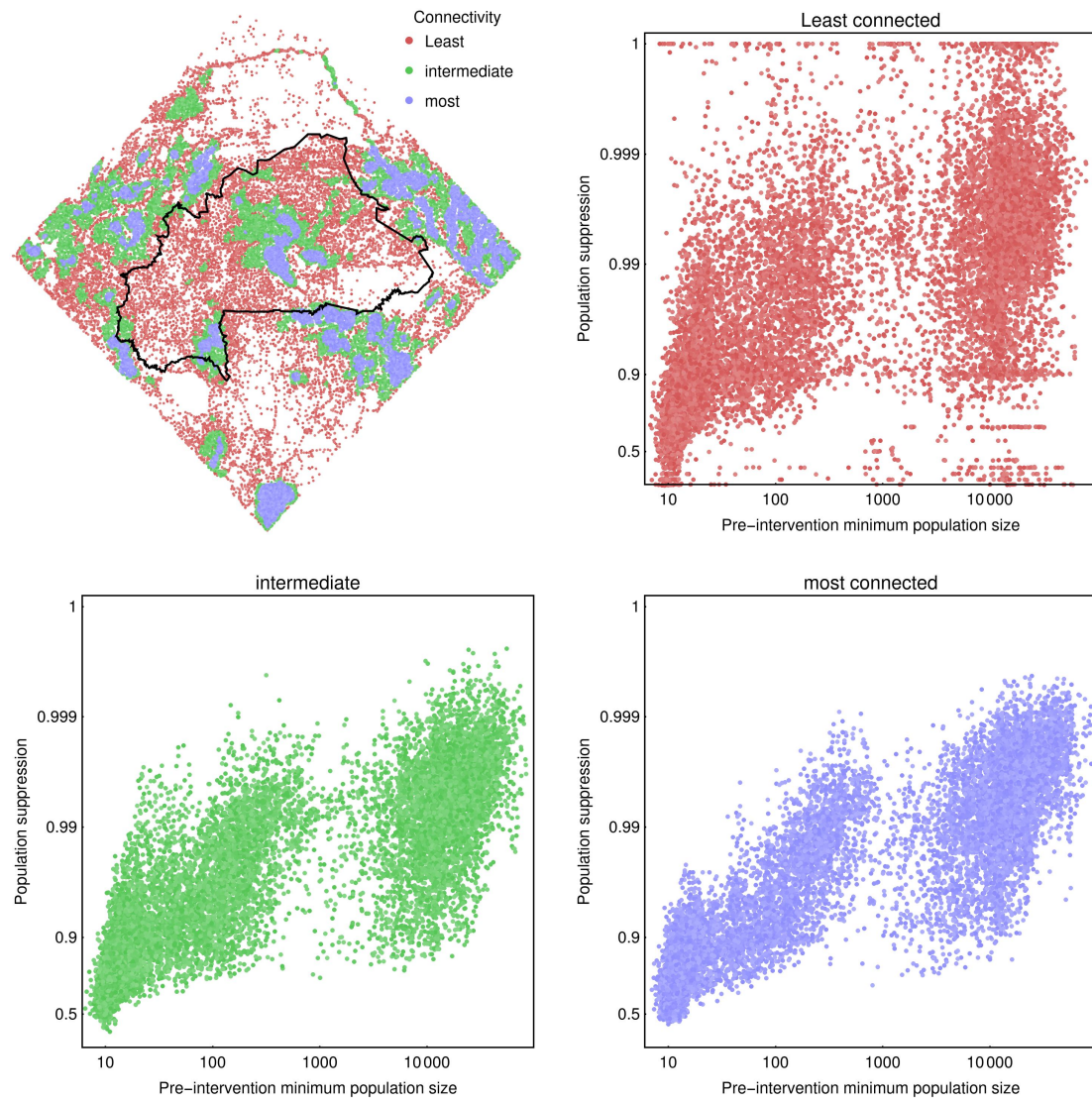

Fig. S5.

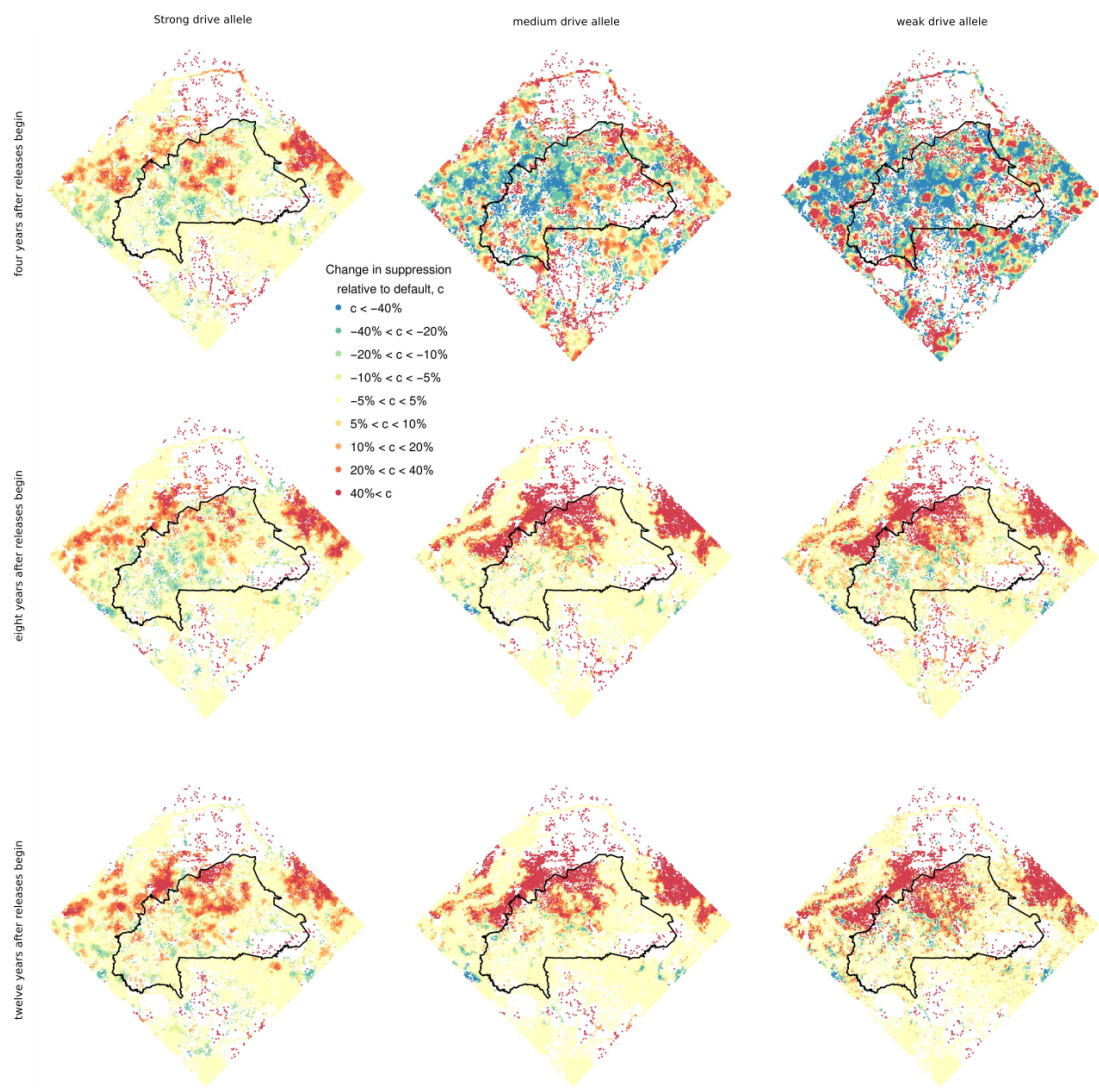

Fig. S6.

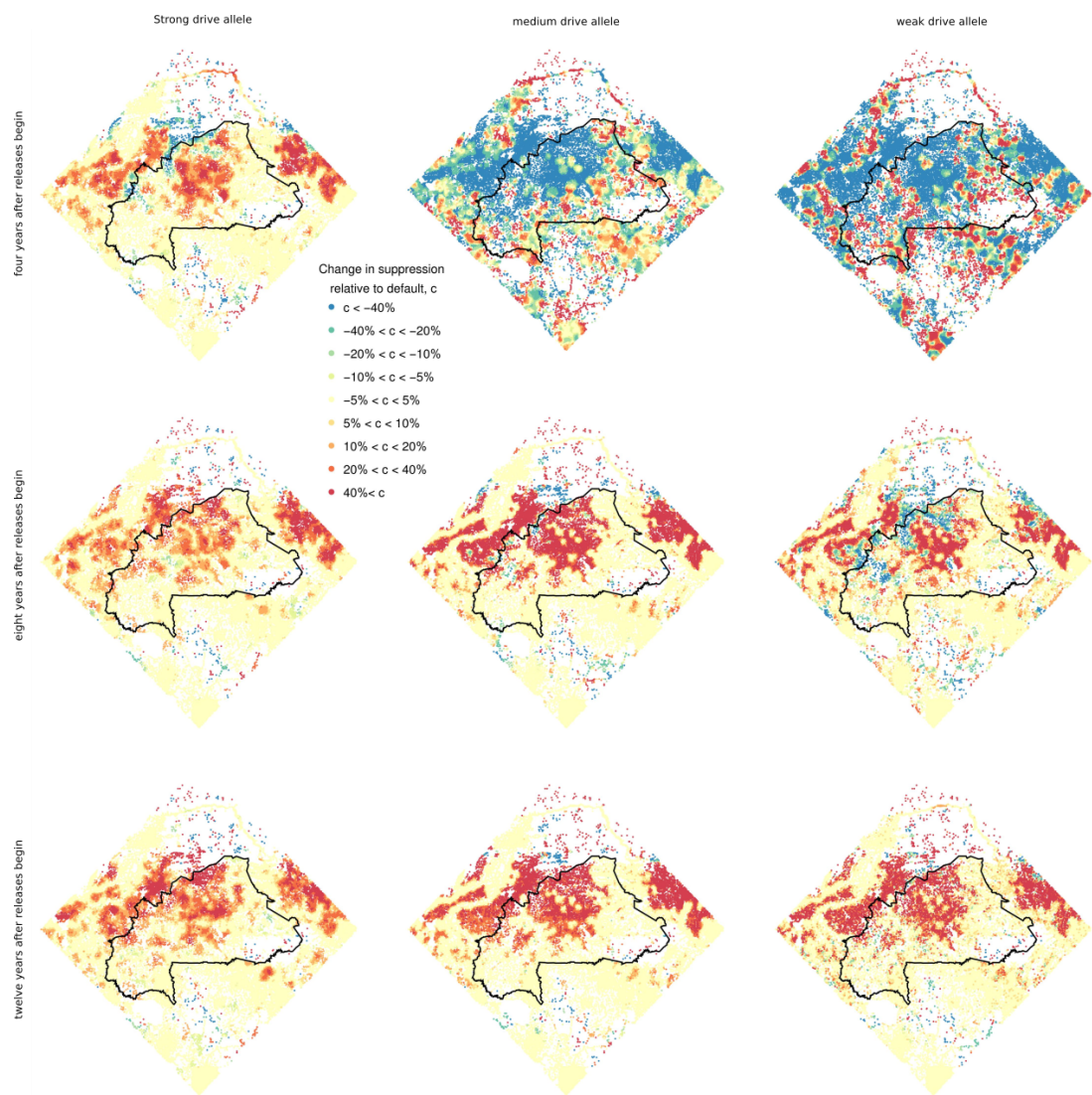

Fig. S7.

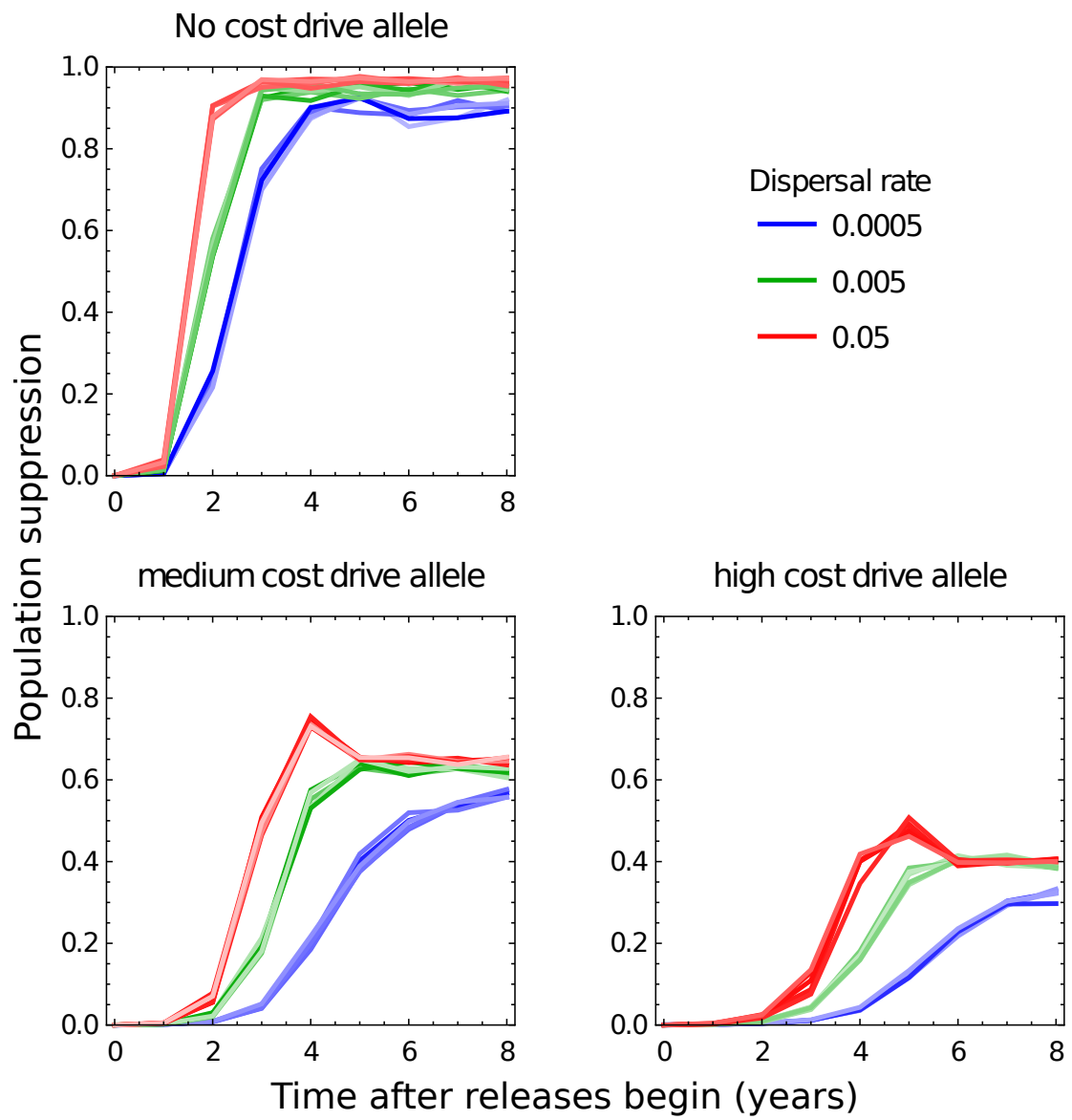

Fig. S8.

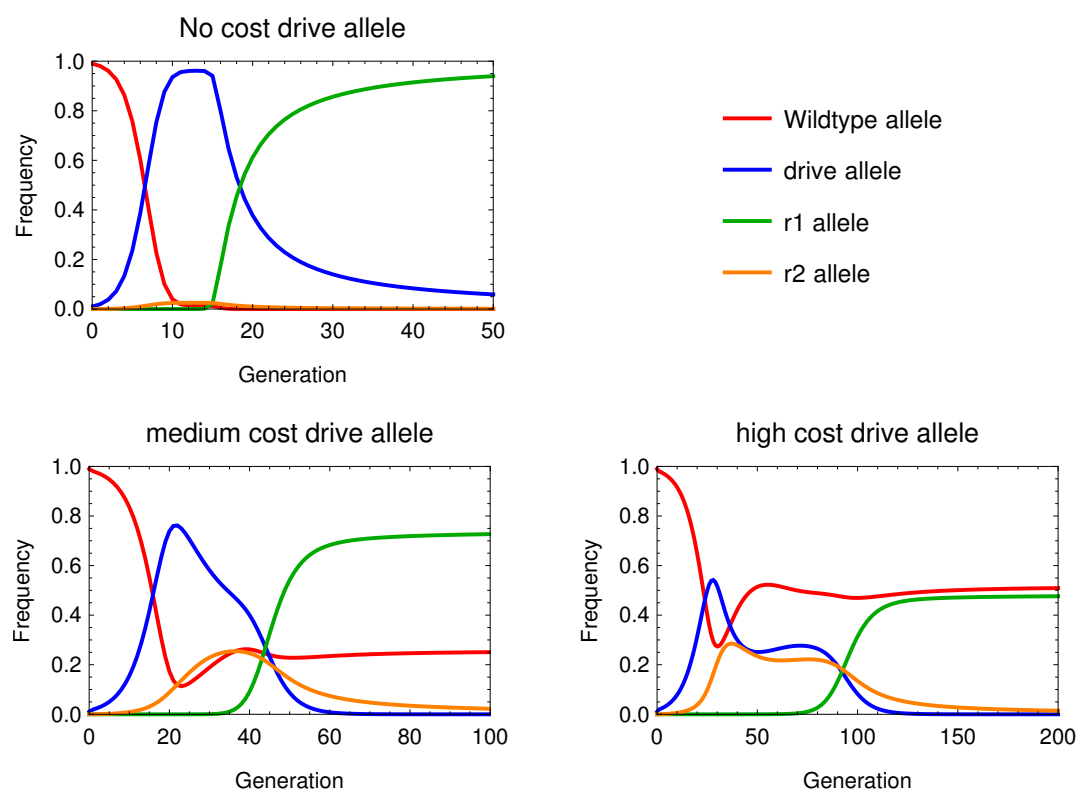

Supplement: Supplementary file 1 — Additional file 1: Figures S1-S8. Fig. S1. Simulated population suppression from the spatial model after four and eight years of releases of the “ideal” drive allele, depending on the number of release sites per year and the size of the releases. Fig. S2. The non-spatial model shows a high degree of sensitivity to high Cas9 deposition costs, due to alternative equilibria being attractive depending on the precise parameters. In both panels, both and maternal parameters are equal, and we assume there are no somatic expression costs. Fig. S3. If the Cas9 deposition costs are close to, yet below, the threshold for converging to a high drive allele equilibrium (cf. Fig. S2), the genetic load on the population will also be high at equilibrium. However, the convergence to equilibrium is faster if these costs are not present (dashed lines). Fig S4. The joint influence of connectivity and seasonality on average population suppression. Connectivity of a site is defined as the number of neighbouring sites within a radius of 12km, and the three plotted connectivity levels are the tertiles of this measure across the study area. Fig. S5. Showing how the predictions change if mosquito populations are maintained by frequent long-distance migration rather than by small bodies of permanent water, which was the default assumption. (Figure 3. in the paper plots the spatial variance in suppression eight years after releases begin for the default case, thus corresponding to the middle row here). Fig. S6. As Fig. S5, but now assuming mosquito populations are maintained by adult female aestivation. Fig. S7. The transient dynamics of suppression for the three strengths of drive allele (cf. Fig. 2) and for three rates of dispersal. Fig. S8. Allele frequency dynamics predicted by a version of the non-spatial model that includes fully functional r1 alleles as well as non-functional r2 alleles discussed elsewhere in the text. The three drive alleles differ in somatic expression costs (cf. fig. [file 12915_2020_834_MOESM1_ESM.pdf]
