# Supplementary material for: Modelling the suppression of a malaria vector using a CRISPR-Cas9 gene drive to reduce female fertility
Source: BMC Biol. 2020 Aug 11;18:98. doi: 10.1186/s12915-020-00834-z (PMC7422583; doi:10.1186/s12915-020-00834-z)
Supplement: Supplementary file 2 — Additional file 2: Table S1. The mean, minimum, and maximum predictions of population suppression depending on the dry season ecology and the drive allele strength. Each result is from ten simulation runs. [file 12915_2020_834_MOESM2_ESM.pdf]

Dry season ecology	Drive allele type	Mean	Min	Max
permanent water	no cost	0.957624	0.947303	0.970865
aestivation	no cost	0.978154	0.967775	0.983998
migration	no cost	0.974715	0.943551	0.988856
permanent water	medium cost	0.623129	0.617146	0.630855
aestivation	medium cost	0.684563	0.676669	0.702339
migration	medium cost	0.671924	0.654531	0.690659
permanent water	high cost	0.392653	0.382462	0.404448
aestivation	high cost	0.393705	0.388605	0.403217
migration	high cost	0.420825	0.408752	0.426442

Table 1: Additional file S2: Table S1
