# Supplementary material for: Modelling the suppression of a malaria vector using a CRISPR-Cas9 gene drive to reduce female fertility
Source: BMC Biol. 2020 Aug 11;18:98. doi: 10.1186/s12915-020-00834-z (PMC7422583; doi:10.1186/s12915-020-00834-z)
Supplement: Supplementary file 3 — Additional file 3. Equations of the population genetics that define the non-spatial model and underlie the spatial model. [file 12915_2020_834_MOESM3_ESM.pdf]

Non-spatial model: Population genetics of a drive allele targeting the *doublesex* gene

Genetic model

We denote by 'H' the homing allele, which is inserted at a locus normally occupied by the wildtype allele 'W' (the *doublesex* gene). Further, non-functional mutant alleles R (that are resistant to homing) may establish at this locus from NHEJ (or MMEJ) events. Though we expect many variants of non-functional resistant alleles would arise in response to the homing allele, we assume these variations are synonomous, and we thus treat the ensemble as a single entity.

The wildtype gene is essential for female fertility so that H/H, R/R, and H/R females are fully sterile, while wildtype heterozygous females have possibly reduced fertility (see below). Males are unaffected by genotype.

Nuclease expression

Homing

We suppose that adults (both male and female) with the genotype W/H create gametes at meiosis in the ratio $\theta_i : e'_i : \gamma'_i$ of $W : H : R$, where i is sex (m or f) and

$$\begin{aligned}
\theta_i &= \frac{1-e_i}{2}(1-\gamma_i) \\
e'_i &= \frac{1+e_i}{2} \\
\gamma'_i &= \frac{1-e_i}{2}\gamma_i
\end{aligned}$$

Here e_i is the sex-specific 'homing rate' and γ_i is the 'NHEJ rate' that corresponds to the creation of R alleles by an NHEJ (or MMEJ) event during meiosis.

Paternal male bias

We wish to study two types of female sterility drive alleles. First, the 'standard' version reduces female fertility as described above but has no effect on sex-ratio (in the manner of Kyrou et al. (2018)). The second type has similar effects on female fertility, and in addition expresses 'paternal male bias', whereby the sex-ratio of offspring sired by male carriers is male-biased (in the manner of Simoni et al., 2020). To incorporate both types into the same model, we define the parameter b to be the extent of male bias, so that the male:female sex-ratio of offspring of W/H and H/H males is $b^+ : b^-$ where

$$\begin{aligned}
b^+ &= \frac{1+b}{2} \\
b^- &= \frac{1-b}{2}.
\end{aligned}$$

In this formulation, the no sex-biasing type of construct is recovered by setting $b = 0$.

Fitness costs

Though the gene-drive construct targeting doublesex is intended to be recessive, Kyrou et al. (2018) found that the fertility of females may be affected by ectopic expression of the nuclease in somatic cells and/or parental deposition of the cas9 nuclease into the newly fertilized embryos. To account for these possibilities we calssify adult females by both their genotype (W/W, W/H, or H/H), and whether one or both parents carried the homing allele. There are nineteen possible types of adult female:

$$(\{F^q_{ww}\}_{q\in\{n,m,f,b\}}, \{F^q_{wh}\}_{q\in\{m,f,b\}}, F_{hh}, \{F^q_{wr}\}_{q\in\{n,m,f,b\}}, \{F^q_{rr}\}_{q\in\{n,m,f,b\}}, \{F^q_{hr}\}_{q\in\{m,f,b\}})$$

where the subscript is genotype and superscript indicates a parent that carried the homing allele (neither, n, male, m, female, f, or both, b). We will consider the four classes $\{F^q_{rr}\}_{q\in\{n,m,f,b\}}$ and the three classes $\{F^q_{hr}\}_{q\in\{m,f,b\}}$ as one (F_{rr} and F_{hr}), since we assume these are all sterile. The parental affects may alter fitness in all other cases, and we thus have 14 classes to consider:

$$(\{F^q_{ww}\}_{q\in\{n,m,f,b\}}, \{F^q_{wh}\}_{q\in\{m,f,b\}}, F_{hh}, \{F^q_{wr}\}_{q\in\{n,m,f,b\}}, F_{rr}, F_{hr}).$$

We denote the fitness cost of ectopic expression of the homing allele in somatic cells to be ξ, and suppose this affects all W/H type females. We denote the fitness cost of maternal and paternal deposition of cas9 to be ρ_f and ρ_m respectively. We assume these costs are multiplicative, giving the vector of female fitnesses:

$$\begin{pmatrix} \omega^n_{ww} \\ \omega^m_{ww} \\ \omega^f_{ww} \\ \omega^b_{ww} \\ \omega^m_{wh} \\ \omega^f_{wh} \\ \omega^b_{wh} \\ \omega_{hh} \\ \omega^n_{wr} \\ \omega^m_{wr} \\ \omega^f_{wr} \\ \omega^b_{wr} \\ \omega_{rr} \\ \omega_{hr} \end{pmatrix} = \text{Fitness} \begin{pmatrix} F^n_{ww} \\ F^m_{ww} \\ F^f_{ww} \\ F^b_{ww} \\ F^m_{wh} \\ F^f_{wh} \\ F^b_{wh} \\ F_{hh} \\ F^n_{wr} \\ F^m_{wr} \\ F^f_{wr} \\ F^b_{wr} \\ F_{rr} \\ F_{hr} \end{pmatrix} = \begin{pmatrix} 1 \\ (1-\rho_m) \\ (1-\rho_f) \\ (1-\rho_m)(1-\rho_f) \\ (1-\xi)(1-\rho_m) \\ (1-\xi)(1-\rho_f) \\ (1-\xi)(1-\rho_m)(1-\rho_f) \\ 0 \\ 1 \\ (1-\rho_m) \\ (1-\rho_f) \\ (1-\rho_m)(1-\rho_f) \\ 0 \\ 0 \end{pmatrix} \quad (1)$$

Since males are unaffected by parental effects, we only need consider six adult types:

$$(M_{ww}, M_{wh}, M_{hh}, M_{wr}, M_{rr}, M_{hr}).$$

We assume all males are equally fit.

Derivation of model equations

First, we separately derive the frequencies of all sperm and ovum gametes that form the next generation. We write sperm gametic frequencies as

$$(M^X_w, M^Y_w, M^{X\dagger}_w, M^{Y\dagger}_w, M^{X\dagger}_h, M^{Y\dagger}_h, M^X_r, M^Y_r, M^{X\dagger}_r, M^{Y\dagger}_r),$$

where the superscript X or Y denotes the sex-chromosome and † indicates the sperm comes from a homing allele bearing father. Similarly we write ovum gametic frequencies as

$$(F_w, F^\dagger_w, F^\dagger_h, F_r, F^\dagger_r).$$

We have

$$\text{Sperm gametic frequencies:}\quad \begin{cases} M_w^X : M_{ww}/2 + M_{wr}/4 \\ M_w^Y : M_{ww}/2 + M_{wr}/4 \\ M_w^{X\dagger} : b^-\theta_m M_{wh} \\ M_w^{Y\dagger} : b^+\theta_m M_{wh} \\ M_h^X : b^- e'_m M_{wh} + b^- M_{hh} + b^- M_{hr}/2 \\ M_h^Y : b^+ e'_m M_{wh} + b^+ M_{hh} + b^+ M_{hr}/2 \\ M_r^X : M_{wr}/4 + M_{rr}/2 \\ M_r^Y : M_{wr}/4 + M_{rr}/2 \\ M_r^{X\dagger} : b^-\gamma'_m M_{wh} + b^- M_{hr}/2 \\ M_r^{Y\dagger} : b^+\gamma'_m M_{wh} + b^+ M_{hr}/2 \end{cases}$$

$$\text{Ovum relative gametic frequencies:}\quad \begin{cases} F_w : (F_{ww}^n + \omega_{ww}^m F_{ww}^m + \omega_{ww}^f F_{ww}^f + \omega_{ww}^b F_{ww}^b) \\ +(F_{wr}^n + \omega_{wr}^m F_{wr}^m + \omega_{wr}^f F_{wr}^f + \omega_{wr}^b F_{wr}^b)/2 \\ F_w^\dagger : \theta_f(\omega_{wh}^m F_{wh}^m + \omega_{wh}^f F_{wh}^f + \omega_{wh}^b F_{wh}^b) \\ F_h : e'_f(\omega_{wh}^m F_{wh}^m + \omega_{wh}^f F_{wh}^f + \omega_{wh}^b F_{wh}^b) \\ F_r : (F_{wr}^n + \omega_{wr}^m F_{wr}^m + \omega_{wr}^f F_{wr}^f + \omega_{wr}^b F_{wr}^b)/2 \\ F_r^\dagger : \gamma'_f(\omega_{wh}^m F_{wh}^m + \omega_{wh}^f F_{wh}^f + \omega_{wh}^b F_{wh}^b) \end{cases}$$

These are converted into next generation adult relative frequencies:

$$\begin{aligned}
F_{ww}^n &= M_w^X F_w \\
F_{ww}^m &= M_w^{X\dagger} F_w \\
F_{ww}^f &= M_w^X F_w^\dagger \\
F_{ww}^b &= M_w^{X\dagger} F_w^\dagger \\
F_{wh}^m &= M_h^{X\dagger} F_w \\
F_{wh}^f &= M_w^X F_h^\dagger \\
F_{wh}^b &= M_w^{X\dagger} F_h^\dagger + M_h^X F_h^\dagger \\
F_{hh} &= M_h^{X\dagger} F_h^\dagger \\
F_{wr}^n &= M_w^X F_r + M_r^X F_w \\
F_{wr}^m &= M_w^{X\dagger} F_r + M_r^{X\dagger} F_w \\
F_{wr}^f &= M_w^X F_r^\dagger + M_r^X F_w^\dagger \\
F_{wr}^b &= M_w^{X\dagger} F_r^\dagger + M_r^{X\dagger} F_w^\dagger \\
F_{rr} &= M_r^X F_r + M_r^{X\dagger} F_r + M_r^X F_r^\dagger + M_r^{X\dagger} F_r^\dagger \\
F_{hr} &= M_h^{X\dagger} F_r + M_r^X F_h^\dagger + M_h^X F_r^\dagger + M_r^{X\dagger} F_h^\dagger \\
M_{ww} &= (M_w^Y + M_w^{Y\dagger})(F_w + F_w^\dagger) \\
M_{wh} &= M_h^{Y\dagger}(F_w + F_w^\dagger) + (M_w^Y + M_w^{Y\dagger})F_h^\dagger \\
M_{hh} &= M_h^{Y\dagger} F_h^\dagger \\
M_{wr} &= (M_w^Y + M_w^{Y\dagger})(F_r + F_r^\dagger) + (M_r^Y + M_r^{Y\dagger})(F_w + F_w^\dagger) \\
M_{rr} &= (M_r^Y + M_r^{Y\dagger})(F_r + F_r^\dagger) \\
M_{hr} &= M_h^{Y\dagger}(F_r + F_r^\dagger) + (M_r^Y + M_r^{Y\dagger})F_h^\dagger
\end{aligned}$$

Finally, these are converted to genotype frequencies through division by their total.

References

Kyrou, K., Hammond, A. M., Galizi, R., Kranjc, N., Burt, A., Beaghton, A. K., Nolan, T., and Crisanti, A. (2018). A CRISPR-Cas9 gene drive targeting doublesex causes complete population suppression in caged *Anopheles gambiae* mosquitoes. *Nat Biotechnol*, 36:1062.

Simoni, A., Hammond, A. M., Beaghton, A. K., Galizi, R., Taxiarchi, C., Kyrou, K., Meacci, D., Gribble, M., Morselli, G., Burt, A., et al. (2020). A male-biased sex-distorter gene drive for the human malaria vector anopheles gambiae. *Nature Biotechnology*, pages 1–7.
